# A Biomechanical Stability Study of Extraforaminal Lumbar Interbody Fusion on the Cadaveric Lumbar Spine Specimens

**DOI:** 10.1371/journal.pone.0168498

**Published:** 2016-12-22

**Authors:** Song Guo, Cheng Zeng, Meijun Yan, Yingchao Han, Dongdong Xia, Guixin Sun, Lijun Li, Mingjie Yang, Jun Tan

**Affiliations:** 1 Department of Spine, Shanghai East Hospital, Tongji University School of Medicine, Shanghai, China; 2 Department of Traumatology, Shanghai East Hospital, Tongji University School of Medicine, Shanghai, China; Mayo Clinic Minnesota, UNITED STATES

## Abstract

**Background:**

Transforaminal lumbar interbody fusion (TLIF) is an effective surgery for lumbar degenerative disease. However, this fusion technique requires resection of inferior facet joint to provide access for superior facet joint resection, which results in reduced lumbar spinal stability and unnecessary trauma. We have previously developed extraforaminal lumbar interbody fusion (ELIF) that can avoid back muscle injury with direct nerve root decompression. This study aims to show that ELIF enhances lumbar spinal stability in comparison to TLIF by comparing lumbar spinal stability of L4–L5 range of motion (ROM) on 12 cadaveric spine specimens after performing TLIF or ELIF.

**Methods:**

12 cadaveric spine specimens were randomly divided and treated in accordance with the different internal fixations, including ELIF with a unilateral pedicle screw (ELIF+UPS), TLIF with a unilateral pedicle screw (TLIF+UPS), TLIF with a bilateral pedicle screw (TLIF+BPS), ELIF with a unilateral pedicle screw and translaminar facet screw (ELIF+UPS+TLFS) and ELIF with a bilateral pedicle screw (ELIF+BPS). The treatment groups were exposed to a 400-N load and 6 N·m movement force to calculate the angular displacement of L4-L5 during anterior flexion, posterior extension, lateral flexion and rotation operation conditions.

**Results:**

The ROM in ELIF+UPS group was smaller than that of TLIF+UPS group under all operating conditions, with the significant differences in left lateral flexion and right rotation by 36.15% and 25.97% respectively. The ROM in ELIF+UPS group was higher than that in TLIF+BPS group. The ROM in the ELIF+UPS+TLFS group was much smaller than that in the ELIF+UPS group, but was not significantly different than that in the TLIF+BPS group.

**Conclusions:**

Despite that TLIF+BPS has great stability, which can be comparable by that of ELIF+UPS. Additionally, ELIF stability can be further improved by using translaminar facet screws without causing more tissue damage to patient.

## Introduction

Lumbar degenerative disease is the leading cause of lower back pain and disability around the world [1–3].The medical conditions of this disease are presented as disc herniation, lumbar spinal canal stenosis, facet joint arthropathy or a combination. There is a broad spectrum of treatment options available including both conservative and operative approaches. A systematic review by Phillips *et*. *al*. [4] concludes that lumbar spinal fusion is an effective treatment strategy for patients who are refractory to non-surgical treatment. A variety of surgical techniques have been developed for lumbar spinal fusion [5–9], of which transforaminal lumbar interbody fusion (TLIF) with bilateral pedicle screw (BPS) fixation has been considered as the classical surgical approach. The benefits of TLIF include increased fusion surface area, restoration of intervertebral body height and lumbar lordosis [10–12]. However, TLIF adopts a posterior approach that leads to decreased soft-tissue mobility [13,14]. A number of new fusion techniques such as anterior lumbar interbody fusion (ALIF), direct lumbar interbody fusion (DLIF) and oblique lumbar interbody fusion (OLIF) have been developed in order to reduce back muscle injury related complications. Nevertheless, none could achieve comparable outcome of nerve root decompression than that of TLIF, since direct decompression in lateral spinal canal cannot be performed through those surgical approaches [15, 16]. In previous study, we designed a new fusion technique named extraforaminal lumbar interbody fusion (ELIF) that could avoid back muscle injury and directly decompress nerve root [17–19]. However, the biomechanical stability of ELIF was not tested. TLIF surgery requires resection of inferior facet joint to provide access for superior facet joint resection. However, inferior facet joint in most cases do not cause spinal canal stenosis. Resection of inferior facet joint reduces lumbar spinal stability and leads to unnecessary trauma. In ELIF surgery, only superior facet joint is resected both inferior facet joint and soft tissues attached behind it are retained. Therefore, we hypothesize that ELIF surgery can provide better lumbar stability than that of TLIF surgery. We analyze the biomechanical stability of this new technique under different internal fixation conditions was analyzed on fresh cadaveric lumbar spines.

## Materials and Methods

### Ethic approval

This study was reviewed and approved by the ethics committee at Shanghai East Hospital, Tongji University School of Medicine. The experiment was performed in accordance with Declaration of Helsinki related to research carried out on human subjects. All donors and the next of kin were fully legally competent and consented to the use of spine for research. Written informed consent from the donor or the next of kin was obtained. None of the donors were from a vulnerable population and all donors or next of kin had provided written informed consent that was freely given. All family dependents were notified and written informed consents were obtained before lumbar spines harvested. The privacy rights of the family dependents were always observed. The next of kin of the individual in this manuscript has given written informed consent to publish case details.

### Sample collection and embedding

Twelve fresh-frozen cadavers were collected from April to August, 2015 (S1 Table). All the cadavers were frozen within six hours after death. There were six female and six male specimens. The average age of all the cadavers was 55.2 (45–63) years old. Structural abnormalities and obvious degradation were excluded by X-ray/CT and visual inspection. Samples were sealed and stored in a freezer at -20°C, and taken out 24 h before the experiment. The samples were naturally thawed at room temperature (20–25°C). All muscles around the vertebral body were removed, and integrity of the intervertebral disc, ligaments, small joints, and vertebrae were preserved as much as possible. Polymethyl methacrylate was used on a special embedding device to embed the samples in preparation for the thoracic and lumbar tests.

### Experimental equipment

Pedicle screws (Moss Miami SI series) and translaminar facet screws were both obtained from Depuy Spine, Inc. (Raynham, MA, USA). The pedicle screw dimensions were 6.0 mm*45 mm, and the translaminar facet screw dimensions were 3.5 mm* 45 mm. The dimensions of the interbody fusion cage (Concorde series) were 9mm*11 mm*27 mm. A ZwickBZ2.5/TS1S universal testing machine (maximum loading, 2kN; ZwickRoell Company, Germany), CCD camera (JAI CV-A1, Denmark), the MATFOLT CoDigital imaging software system (developed by the Solid Mechanics Laboratory Center of Shanghai University) and 3D printer (Stratasys 350 Connex 3D, MN, USA) were used.

### Experimental grouping and operation methods

**Fig 1:**
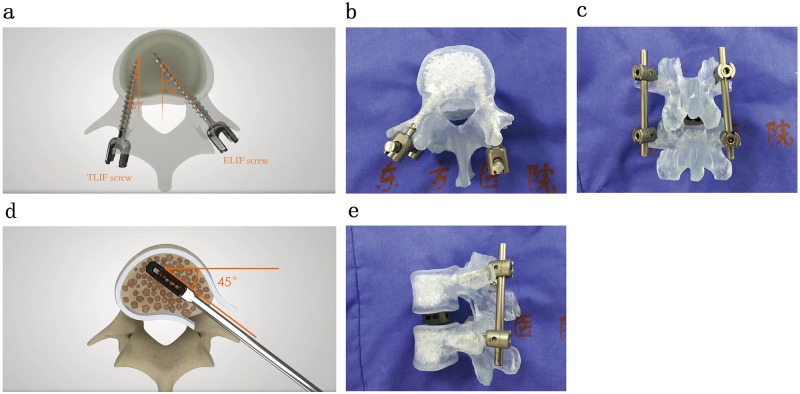
**(panel a- panel c)** These **panel**s indicate the different angle of inserting screw between ELIF and TLIF. In panel (b) and panel (c), a transparent 3D model was used to clearly show the difference of the angle of screw. **(panel d-e)** They indicate that the angle of inserting cage is 45° with the sagittal plane. In panel e, the transparent 3D model is also used to show the angle of inserting cage.

**Fig 2:**
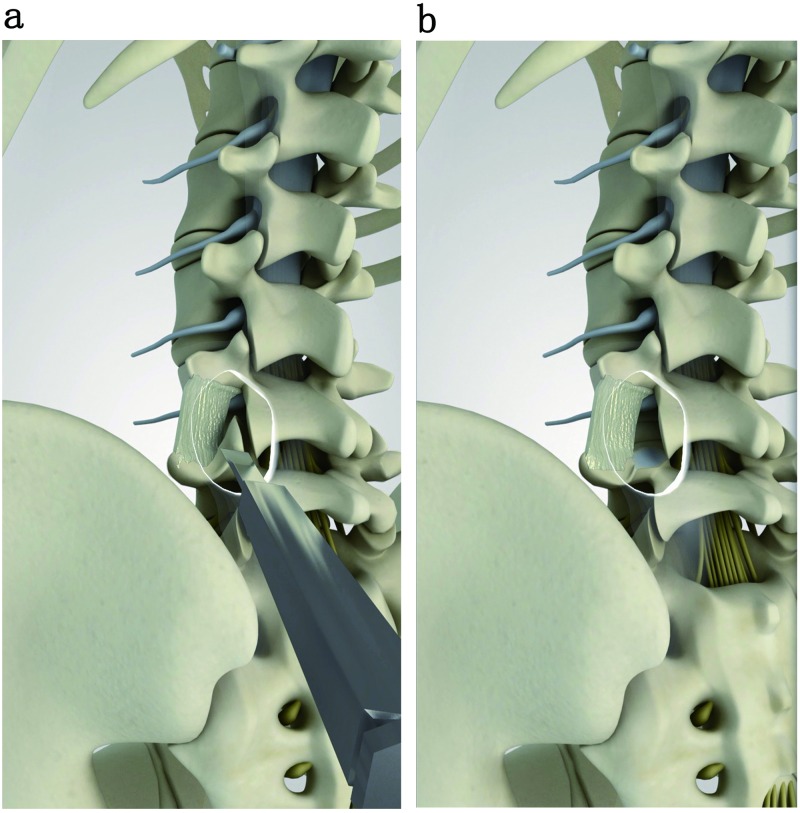
They show that only L5 superior facet joint was removed by ostetome.

Biomechanical tests were conducted on the 12 samples under anterior flexion and posterior extension, left and right lateral flexion, and left and right rotation. Before surgery, the L4-L5 range of motions (ROMs) of all intact samples were obtained, and recorded as the control group. The 12 samples were then randomly divided into two groups: TLIF group and ELIF group. We treated the two groups in accordance with the different internal fixations, including ELIF with a unilateral pedicle screw (ELIF+UPS), TLIF with a unilateral pedicle screw (TLIF+UPS), TLIF with a bilateral pedicle screw (TLIF+BPS), ELIF with a unilateral pedicle screw and translaminar facet screw (ELIF+UPS+TLFS) and ELIF with a bilateral pedicle screw (ELIF+BPS). TLIF surgery was conducted on the samples in the TLIF group based on the classical approach: The right L4 inferior facet joint and L5 superior facet joint were removed. The entire nucleus pulposus in the L4-L5 disc and the right posterior 2/3rds of the fibrous ring were resected, and a cage was implanted. The posterior supraspinous ligament, interspinous ligament, spinous process, and left structures were retained. A screw each was placed in the L4 and L5 pedicles on the right side (the entry point of the screws was the herringbone crest vertex, at a 15° angle with the sagittal plane Fig 1a–1c). ELIF surgery was conducted on the samples in the ELIF group: Only the right L5 superior facet joint was removed by osteotome (Fig 2a and 2b). The entire nucleus pulposus in the L4-L5 disc as well as the right posterior 2/3rds of the fibrous ring were resected, and a cage was implanted at a 45° angle with the sagittal plane (Fig 1d and 1e). The posterior supraspinous ligament, interspinous ligament, spinous process, and left structures were also retained. One screw each was placed in the right L4 and L5 pedicles (the entry point of the screws was the transition point between the superior facet joint and the transverse process, at a 45° angle with the sagittal plane (Fig 1a–1c). The above samples were recorded as TLIF+UPS group and ELIF+UPS group, respectively, which were also the basic groups. For the TLIF+BPS group, additional contralateral pedicle screw fixation was applied via the conventional implantation approach. In the ELIF+UPS+TLFS group, an additional translaminar facet screw was implanted into the contralateral facet joint through the decompression side. For the ELIF+BPS group, additional contralateral pedicle screw fixation was applied via the conventional implantation approach.

### Biomechanical tests

**Fig 3:**
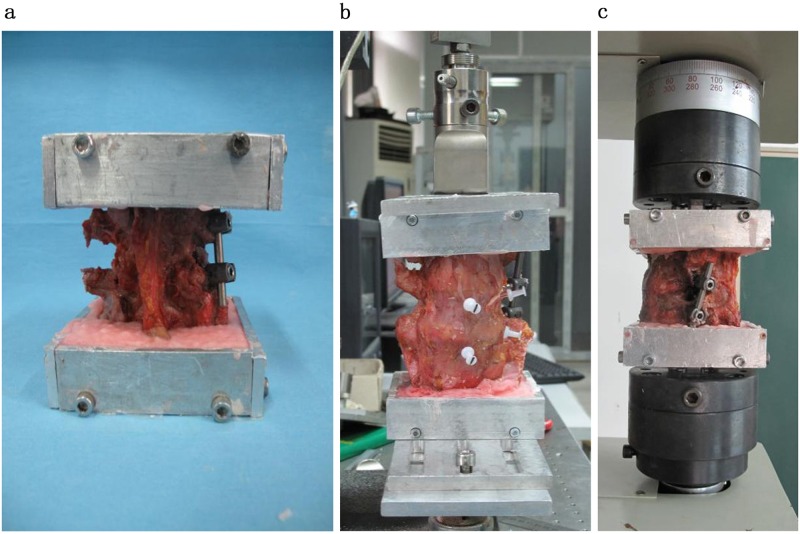
**(panel a)** It shows L5 vertebral was embedded into a box. **(panel b- panel c)** They show the L5 ROMs under six operating conditions were tested on the machine.

Due to limit specimens and high cost, the Panjabi internal fixation stability test method was adopted for the biomechanical tests. This test is a non-destructive method, and repeated tests could be conducted in every group. Metal-labeled screws with marks of 1mm diameter were implanted at 4 points—the most forward point, most backward point, most leftward point, and most rightward point—on the superior surfaces of the L4 and L5 vertebrae. An embedding box containing the L5 vertebral body was placed on a ZwickBZ2.5/TS1S universal testing machine (maximum loading, 2kN; ZwickRoell Company, Germany) (Fig 3a). Surface loading was applied on the superior surface of the L4 vertebral body, in a vertically downward direction and with a uniform distribution over the entire superior endplate of the L4 vertebral body. The load applied on model was 400N; the movement force was 6N·m; and the loading rate was 5mm/min. The sample was tested under 6 operating conditions, namely, lumbar spine anterior flexion (AF), posterior extension (PE), left and right lateral flexion (LF and RF), and left and right rotation (LR and RR) (Fig 3b and 3c). The L4–L5 range of motion (ROM) in different groups was determined using the L4-L5 segmental angular displacement. The spatial coordinates of the 4 points at which the metal screws were placed were determined, and the points were connected with lines. The angles between the lines represented the angles between the superior surfaces of two neighbouring vertebral bodies. The absolute value of the difference in the angles before and after loading was the angular displacement of the L4–L5 segments. A CCD camera (JAI CV-A1, Denmark) was used to collect sequential images of motion-related changes in the lumbar spine in the sagittal and coronal planes, and the MATFOLT Co Digital imaging software system (developed by the Solid Mechanics Laboratory Center of Shanghai University) was used to process the images. During the entire process, 0.9% saline was used to keep the ligaments in the samples moist. The loading tests were conducted on every sample in the following sequence: anterior flexion/posterior extension, left/right lateral flexion, and left/right rotation. The angular displacement changes during lumbar spine movement were recorded to determine the L4–L5 ROM.

### Statistical analysis

SPSS (version 18.0, SPSS, Inc., Chicago, IL, USA) software was used for statistical analysis. Count data were expressed as mean±standard deviation. All the data were tested for normality with Shapiro-Wilk test. Comparisons between multiple sets of sample means were conducted using one way analysis of variance (ANOVA). P<0.05 was considered statistically significant. If the ROMs were statistically significant, the ROMs for each two group were compared by using post-hoc Tukey’s-multiple-comparisons-test.

## Results and Discussion

### General observations

Internal fixation was stable and reliable in all the lumbar spine samples. There were no cases of fracture displacement, subsidence deformation, etc., at the screw–bone interface and the implantation site of the intervertebral fusion cage. During the entire test, the joint capsule, ligaments, and retained fibrous ring tissues were kept moist and naturally elastic. There were no cases of tearing, breakage, etc.

### Biochemical testing

**Fig 4:**
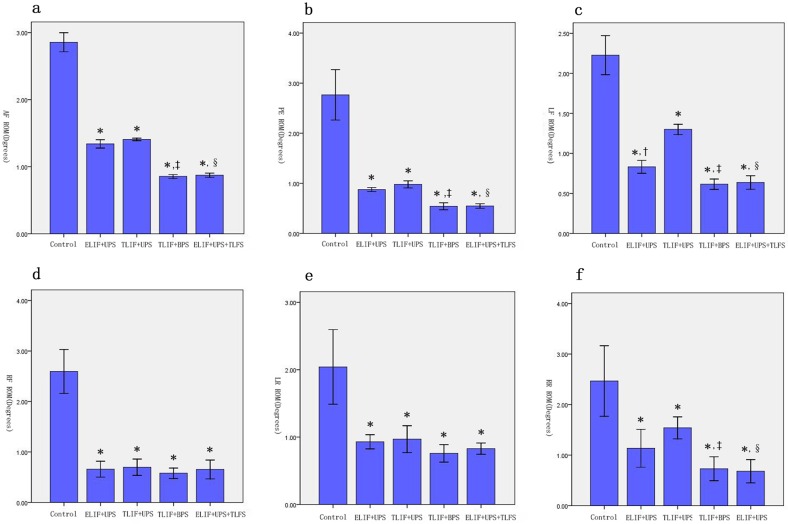
The L4–L5 range of motion (ROM) in different groups under six operating conditions. The ROMs were significantly lower in all the internal fixation groups than the ROMs in the control group. Additionally, the ROM was smaller in the ELIF+UPS group than in the TLIF+UPS group under various operating conditions, and the difference was significant during left lateral flexion and right rotation P<0.05 **(panel c and panel f).** TLIF+BPS group showed statistically smaller ROM than ELIF+UPS group during anterior flexion, posterior extension, left lateral flexion and right rotation P<0.05 **(panel a, panel b, panel c, and panel f).** Similarly, in the ELIF+UPS+TLFS group, the ROMs were much smaller than those in the ELIF+UPS group during anterior flexion, posterior extension, left lateral flexion and right rotation P<0.05 **(panel a, panel b, panel c, and panel f).** There were no statistic difference between ELIF+UPS+TLFS group and TLIF+BPS group. All the data were tested for normality with Shapiro-Wilk test. Comparisons between multiple sets of sample means were conducted using one way analysis of variance (ANOVA). If the ROMs were statistically significant, the each subgroup data were compared using post-hoc Tukey’s-multiple-comparisons-test. P<0.05 was considered statistically significant. *: Internal fixation groups vs control group †: ELIF+UPS group vs TLIF+UPS group ‡: TLIF+BPS group vs ELIF+UPS group §: ELIF+UPS+TLFS group vs ELIF+UPS group ELIF: extraforaminal lumbar interbody fusion; UPS: unilateral pedicle screw TLIF: transforaminal lumbar interbody fusion; BPS: bilateral pedicle screw TLFS: translaminar facet screw AF: anterior flexion; PE: posterior extension; LF: lateral flexion; RF: right flexion; LR: left rotation RR: right rotation.

The L4–L5 ROM in different groups was showed in (Table 1, Fig 4). The ROMs between the five groups were significantly different for every operation condition (p<0.05). Afterwards, the comparison between each two group were performed by using post-hoc Tukey’s-multiple-comparisons-test. The ROMs of all operation conditions were obviously lower in all internal fixation groups than control group (p<0.05). Moreover, the ROM was smaller in the ELIF+UPS group than in the TLIF+UPS group under every operating condition, and the difference was significant during left lateral flexion and right rotation (Fig 4c and 4f P<0.05). During left lateral flexion and right rotation, the ROMs of ELIF+UPS decreased by 36.15% (0.83±0.08 vs 1.30±0.06, P<0.05) and 25.97% (1.14±0.36 vs 1.54±0.21, P<0.05) respectively (Table 1). TLIF+BPS group showed smaller ROM than ELIF+UPS group in all operating conditions, and the percentages of decrease were anterior flexion: 35.82% (0.86±0.03 vs 1.34±0.06, P<0.05), posterior extension: 38.63% (0.54±0.07 vs 0.88±0.04, P<0.05), left lateral flexion: 25.30% (0.62±0.06 vs 0.83±0.08, P<0.05), right lateral flexion: 12.12% (0.58±0.10 vs 0.66±0.15, P>0.05) left rotation: 18.28% (0.76±0.12 vs 0.93±0.10, P>0.05), and right rotation: 35.96% (0.73±0.26 vs 1.14±0.36, P<0.05) (Table 1). Similarly, in the ELIF+UPS+TLFS group, the ROMs were much smaller than those in the ELIF+UPS group (Table 1, Fig 4). The percentages of decrease were anterior flexion: 35.07% (0.87±0.03 vs 1.34±0.06, P<0.05), posterior extension: 37.50% (0.55±0.04 vs 0.88±0.04, P<0.05), left lateral flexion: 22.89% (0.64±0.08 vs 0.83±0.08, P<0.05), right lateral flexion: 1.52% (0.65±0.18 vs 0.66±0.15, P>0.05), left rotation: 10.758% (0.83±0.08 vs 0.93±0.10, P>0.05) and right rotation: 40.35% (0.68±0.22 vs 1.14±0.36, P<0.05) (Table 1). Moreover, the ROMs of ELIF+UPS+TLFS group were similar to those in the TLIF+BPS group, within the limited range of loading. There was no significant difference in ROMs between the ELIF+UPS+TLFS, ELIF+BPS and TLIF+BPS groups under the six operating conditions within the range of experimental loading (S2 Table).

**Table 1:** The L4–L5 range of motion (ROM) in different groups under six operating conditions were compared by using one way analysis of variance.

Groups	AF (°)	PE (°)	LF (°)	RF (°)	LR (°)	RR (°)
Control	2.86±0.14	2.77±0.48	2.23±0.23	2.60±0.42	2.04±0.53	2.47±0.27
ELIF+UPS	1.34±0.06	0.88±0.04	0.83±0.08	0.66±0.15	0.93±0.10	1.14±0.36
TLIF+UPS	1.41±0.02	0.98±0.07	1.30±0.06	0.70±0.16	0.97±0.19	1.54±0.21
TLIF+BPS	0.86±0.03	0.54±0.07	0.62±0.06	0.58±0.10	0.76±0.12	0.73±0.26
ELIF+UPS+TLFS	0.87±0.03	0.55±0.04	0.64±0.08	0.65±0.18	0.83±0.08	0.68±0.22
Percentage decrease from Control to ELIF+UPS	53.15% **P<0.05**	68.23% **P<0.05**	62.78% **P<0.05**	74.62% **P<0.05**	54.41% **P<0.05**	53.85% **P<0.05**
Percentage decrease from Control to TLIF+UPS	50.70% **P<0.05**	64.62% **P<0.05**	41.70% **P<0.05**	73.08% **P<0.05**	52.45% **P<0.05**	37.65% **P<0.05**
Percentage decrease from TLIF+UPS to ELIF+UPS	4.96% P = 0.140	10.20% P = 0.123	36.15% **P<0.05**	5.71% P = 0.177	4.12% P = 0.606	25.97% **P<0.05**
Percentage decrease from ELIF+UPS to TLIF+BPS	35.82% **P<0.05**	38.63% **P<0.05**	25.30% **P<0.05**	12.12% P = 0.055	18.28% P = 0.052	35.96% **P<0.05**
Percentage decrease from ELIF+UPS to ELIF+UPS+TLFS	35.07% **P<0.05**	37.50% **P<0.05**	22.89% **P<0.05**	1.52% P = 0.55	10.75% P = 0.07	40.35% **P<0.05**
Percentage decrease from ELIF+UPS+TLFS to TLIF+BPS	1.16% P = 0.823	1.85% P = 0.870	-5.88% P = 0.808	3.33% P = 0.089	8.43% P = 0.334	-6.85% P = 0.720

ELIF: extraforaminal lumbar interbody fusion; UPS: unilateral pedicle screw

TLIF: transforaminal lumbar interbody fusion; BPS: bilateral pedicle screw

TLFS: translaminar facet screw

AF: anterior flexion; PE: posterior extension; LF: lateral flexion; RF: right flexion; LR: left rotation RR: right rotation.

Bold types indicate statistically significant difference among the different internal fixation groups under the six operating conditions.

Lumbar interbody fusion technique includes three main parts: decompression, fixation, and fusion [20–22]. Decompression is the key step to relieve the patient’s symptoms with nerve root compression. Fixation is used to achieve immediate postoperative stability, which facilitates early rehabilitation and prevents the complications of bed rest. Fusion helps ensure good long-term effects. TLIF surgery as the classic fusion technique mainly focuses on decompressing lateral spinal canal. The stenosis of which is mainly caused by a great reduction in intervertebral height and intervertebral disc herniation, especially with relative cohesion and hyperplasia of superior facet joints [23, 24]. In most cases, the change of inferior articular process is not the etiological cause for lateral spinal canal stenosis. However, inferior facet joint needs to be resected firstly with TLIF in order to achieve the purpose of nerve root decompression. The only purpose of resecting inferior facet joint is to provide access for the resection of superior facet joint. Therefore, the resection of inferior facet joint reduces lumbar spinal stability to some degree and leads to unnecessary trauma. Numerous biomechanical studies have led to the following consensus: Retention of the integrity of the posterior structures, as much as possible, can significantly decrease the risk of adjacent lumbar segments degeneration, increase immediate postoperative stability of the lumbar spine, and improve the fusion rate of the surgical segments [25]. In our previous study, we develop a less invasive and more efficient alternative to conventional TLIF surgery, and namely ELIF. The difference between ELIF and TLIF is that ELIF completely retains inferior facet joint on the surgical side and the joint capsule, ligaments, muscles, and other tension band structures that are attached behind it. ELIF is a clinical improvement that fully absorbs all the advantages of TLIF, is less invasive than TLIF, and more completely maintains the posterior structures. The special entry point (opening 6–9 cm beside the midline) and angle (45° oblique to the direction of the vertebral body) of the ELIF surgical approach avoid the inferior facet joint and back muscle, and allow the surgery to be directly focused on superior facet joint. After the resection of superior facet joint, the inner structure inside the intervertebral foramen can be accessed. Through the expanded bony window, the intervertebral disc can be safely resected for appropriate cage placement [17].

The implantation angle of intervertebral fusion cage in ELIF is different with TLIF. The cage is implanted at an angle of 45° with the sagittal plane using ELIF. As compared with conventional TLIF, the direction of intervertebral fusion cage implantation is more oblique; hence, theoretically a longer cage can be placed into intervertebral space. In addition, the entry point of pedicle screw is moved outward and entry angle is more inclined in ELIF. Therefore, it may be feasible to apply a longer pedicle screw fixation in ELIF surgery. It will be tested in the further study in terms of these two points described above. As is known to us, the longer cage placement and longer pedicle screw fixation can both affect the biomechanical stability. In this study, we use the same size of cage and pedicle screw in ELIF group and TLIF group to exclude the effect of different cage and pedicle screw size on biomechanical stability.

In this study, the posterior lumbar stability of ELIF and TLIF under different internal fixation conditions was assessed and compared using biomechanical testing. The analyses indicated that spinal stability was better in the case of ELIF with a unilateral pedicle screw fixation than in the case of TLIF with a unilateral pedicle screw fixation, under various operating conditions, especially, left lateral flexion and right rotation. The reason for this finding may be that in ELIF+UPS group, part of superior facet joint retained on the decompression side could associate with inferior facet joint to limit left lateral flexion and right rotation of the lumbar spine, and improve spinal stability. Additionally, the screws in the ELIF+UPS group had greater extraversion, and the direction in which the cage was implanted was closer to the coronal plane. Both were also beneficial to improve lumbar spine stability. However, the stability of ELIF with unilateral pedicle screw fixation was significantly weaker than that of the TLIF with bilateral pedicle screw fixation, indicating that ELIF with a unilateral pedicle screw fixation still could not reach the stability achieved by TLIF with a bilateral pedicle screw fixation.

Translaminar facet screw (TLFS) fixation was first introduced in 1948 and developed to improve the lumbar stability [26]. Recently, this screw fixation technique was applied to lumbar interbody fusion. Some studies showed that unilateral pedicle screw fixation supplemented with contralateral facet screw fixation could achieve the stability comparable to bilateral pedicle screws [27–29]. Moreover, it was feasible to implant the screw with only a contralateral small incision, which reduced the skin incision. Additionally, the translaminar facet screw was much cheaper than pedicle screw [30, 31]. Therefore, based on ELIF with a unilateral pedicle screw fixation, an additional translaminar facet screw was implanted into contralateral facet joint from the decompression side, namely ELIF+UPS+TLFS group. The supplemental translaminar facet screw fixation obviously increased the stability as compared to ELIF with a unilateral pedicle screw fixation. Moreover, the stability associated with the former did not significantly differ from that associated with the classic TLIF with a bilateral pedicle screw fixation within the experimental loading range. This result suggested that the stability of ELIF with a unilateral fixation could be improved by an additional screw fixation through contralateral lamina and facet joint.

In summary, ELIF with a unilateral pedicle screw and translaminar facet screw fixation could meet the stability requirements for fusion surgeries [32, 33]. Furthermore, this surgical technique can simultaneously fulfil the decompression, fixation and fusion only with unilateral exposure, which decreasing surgical incision and back muscle injury. Finally, less pedicle screw is used with this technique, which greatly reduces the risk of inserting pedicle screw and surgical expense. This new ELIF surgery can potentially be a stable fusion technique which using a more invasive and economic internal fixation than classical TLIF with bilateral pedicle screw fixation.

Although this study has achieved primary success, more work needs to be done. The cadaver samples proved the feasibility of the surgery, but there are many differences between human bodies and cadavers. Many venous vascular plexuses exist in the intervertebral foraminal region, and injuries to these structures during operation may easily cause bleeding. The ELIF surgery approach is deep, and the surgery directly focuses on the intervertebral foramen. Therefore, attention should be paid to the vascular structures in this area, and hemostasis after bleeding must be achieved during the surgery. What’s more, since the approach differs from the conventional anatomical approach, a learning curve exists for this surgery. Additionally, this surgery involves an inclined direction and a deep approach, and thus, operation with conventional surgical instruments is not convenient. Special surgical instruments need to be designed and tested before the clinical application of ELIF. Finally, this fusion sugrey needs to be further tested in lumbar spondylolisthesis.

## Conclusions

Despite that TLIF+BPS has great stability, which can be comparable by that of ELIF+UPS. Additionally, ELIF stability can be further improved by using translaminar facet screws without causing more tissue damage to patient.

## Supporting Information

S1 Table Details of cadavers. (DOC)

S2 Table The L4–L5 range of motion (ROM) in ELIF+UPS+TLFS, ELIF+BPS and TLIF+BPS under six operating conditions. (DOC)

S1 Fig The written consent from the donors' families to publish the images in Fig 3. (JPG)
