# Supplementary material for: A Biomechanical Stability Study of Extraforaminal Lumbar Interbody Fusion on the Cadaveric Lumbar Spine Specimens
Source: PLoS One. 2016 Dec 22;11(12):e0168498. doi: 10.1371/journal.pone.0168498 (PMC5178989; doi:10.1371/journal.pone.0168498)
Supplement: S1 Table — (DOC) [file pone.0168498.s001.doc]

**S1 Table. Details of cadavers**

Cadavers ID Gender Cause of death Medical history Sources of Cadavers

1 Female Epidural hematoma Healthy Shanghai East Hospital Affiliated to

Tongji University School of Medicine

2 Female Intracranial hemorrhage Healthy Shanghai East Hospital Affiliated to

Tongji University School of Medicine

3 Female Myocardial infarction Hypertension Shanghai East Hospital Affiliated to

Tongji University School of Medicine

4 Male Cerebral infarction Hypertension Shanghai East Hospital Affiliated to

Tongji University School of Medicine

5 Female Epidural hematoma Diabetes Shanghai East Hospital Affiliated to

Tongji University School of Medicine

6 Male Myocardial infarction Diabetes Shanghai East Hospital Affiliated to

Tongji University School of Medicine

7 Male Heart failure Hypertension Shanghai East Hospital Affiliated to

Tongji University School of Medicine

8 Male Renal failure Chronic nephritis Shanghai East Hospital Affiliated to

Tongji University School of Medicine

9 Female Intracranial hemorrhage Healthy Shanghai East Hospital Affiliated to

Tongji University School of Medicine

10 Female Cerebral infarction Hypertension Shanghai East Hospital Affiliated to

Tongji University School of Medicine

11 Male Heart failure COPD Shanghai East Hospital Affiliated to

Tongji University School of Medicine

12 Male Renal failure Chronic nephritis Shanghai East Hospital Affiliated to

Tongji University School of Medicine
