# Supplementary material for: A Biomechanical Stability Study of Extraforaminal Lumbar Interbody Fusion on the Cadaveric Lumbar Spine Specimens
Source: PLoS One. 2016 Dec 22;11(12):e0168498. doi: 10.1371/journal.pone.0168498 (PMC5178989; doi:10.1371/journal.pone.0168498)
Supplement: S2 Table — (DOC) [file pone.0168498.s002.doc]

**S2 Table. The L4–L5 range of motion (ROM) in ELIF+UPS+TLFS, ELIF+BPS and TLIF+BPS under six operating conditions.**

| Groups | AF (°) | PE (°) | LF (°) | RF (°) | LR (°) | RR (°) |
| --- | --- | --- | --- | --- | --- | --- |
| ELIF+UPS+TLFS | 0.87±0.03 | 0.55±0.04 | 0.64±0.08 | 0.60±0.18 | 0.83±0.08 | 0.68±0.22 |
| ELIF+BPS | 0.87±0.04 | 0.58±0.04 | 0.58±0.07 | 0.57±0.07 | 0.77±0.08 | 0.67±0.18 |
| TLIF+BPS | 0.86±0.03 | 0.54±0.07 | 0.62±0.06 | 0.58±0.10 | 0.76±0.12 | 0.73±0.26 |
| Percentage decrease from ELIF+UPS+TLFS to ELIF+BPS | 0.00%  P=0.820 | -5.45% P=0.151 | 9.38%  P=0.140 | 5.00% P=0.074 | 7.22% P=0.174 | 1.47%  P=0.214 |
| Percentage decrease from ELIF+UPS+TLFS to TLIF+BPS | 1.16%  P=0.173 | 1.85%  P=0.786 | -5.88% P=0.643 | 3.33%  P=0.089 | 8.43%  P=0.832 | -6.85%  P=0.169 |
| Percentage decrease from ELIF+BPS  to TLIF+BPS | 1.16%  P=0.173 | 6.90%  P=0.071 | -6.90%  P=0.347 | 1.75%  P=0.420 | 1.30%  P=0.343 | 8.96%  P=0.390 |

ELIF: extraforaminal lumbar interbody fusion; UPS: unilateral pedicle screw

TLIF: transforaminal lumbar interbody fusion; BPS: bilateral pedicle screw

TLFS: translaminar facet screw

AF: anterior flexion; PE: posterior extension; LF: left flexion; RF: right flexion; LR: left rotation RR: right rotation.
