# Supplementary figures and images for: A Biomechanical Stability Study of Extraforaminal Lumbar Interbody Fusion on the Cadaveric Lumbar Spine Specimens
Source: PLoS One. 2016 Dec 22;11(12):e0168498. doi: 10.1371/journal.pone.0168498 (PMC5178989; doi:10.1371/journal.pone.0168498)

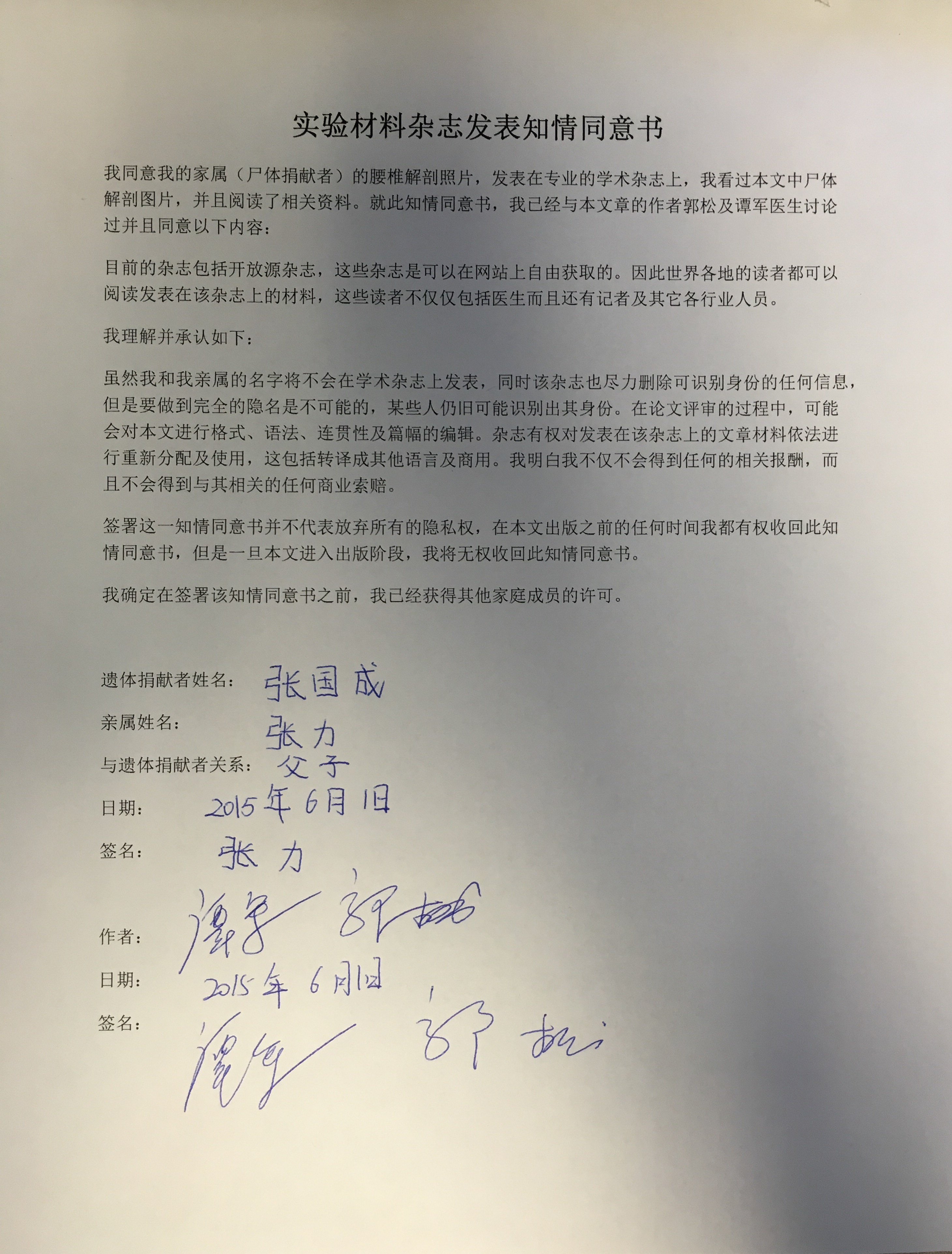

Supplement: S1 Fig — (JPG) [file pone.0168498.s003.JPG]
